# Increasing Cognitive Load Reduces Interference from Masked Appetitive and Aversive but Not Neutral Stimuli

**DOI:** 10.1371/journal.pone.0094417

**Published:** 2014-04-07

**Authors:** Rudolf Uher, Samantha J. Brooks, Savani Bartholdy, Kate Tchanturia, Iain C. Campbell

**Affiliations:** King's College London, Institute of Psychiatry, London, United Kingdom; Center for BrainHealth, University of Texas at Dallas, United States of America

## Abstract

Interactions between cognition and emotion are important for survival, often occurring in the absence of awareness. These interactions have been proposed to involve competition between cognition and emotion for attentional resources. Emotional stimuli have been reported to impair performance on cognitive tasks of low, but not high, load if stimuli are consciously perceived. This study explored whether this load-dependent interference effect occurred in response to subliminal emotional stimuli. Masked emotional (appetitive and aversive), but not neutral, stimuli interfered with performance accuracy but not response time on a cognitive task (n-back) at low (1-back), but not high (2-back) load. These results show that a load-dependent interference effect applies to masked emotional stimuli and that the effect generalises across stimulus categories with high motivational value. This supports models of selective attention that propose that cognition and emotion compete for attentional resources. More specifically, interference from masked emotional stimuli at low load suggests that attention is biased towards salient stimuli, while dissipation of interference under high load involves top-down regulation of attention. Our data also indicate that top-down goal-directed regulation of attention occurs in the absence of awareness and does not require metacognitive monitoring or evaluation of bias over behaviour, i.e., some degree of self-regulation occurs at a non-conscious level.

## Introduction

Our world is replete with emotionally laden stimuli, much of which we have learned to ignore. However, unattended stimuli may still influence our thinking and behaviour, e.g., ‘gut feelings’ are used to make decisions, often to our advantage [1]. Moreover, non-conscious perception of subliminally presented stimuli can affect behaviour [2]. Rapid responses to salient environmental cues, especially those with high motivational value (e.g., appetitive or aversive stimuli) offers an important evolutionary survival advantage [3] but, on the other hand, constant reorientation to salient environmental cues may be detrimental to the maintenance of goal-directed behaviour. It is likely therefore that processes exist that regulate interactions between cognition and emotion [4]. Accordingly, several groups have proposed that emotion and cognition compete for limited attentional resources [5]–[9]. Salient stimuli with high motivational value are likely to capture more attentional resources than those that are less motivationally significant [9] and in a similar way, attentional resources are taxed by increasing task demands (e.g., task difficulty). Thus, in different scenarios, there is a need for either cognition or emotion to dominate. Evidence for such interactions has been provided by demonstrations of interference (impaired performance) resulting from the presentation of distracting emotional stimuli during cognitive tasks, and in a similar way, cognitive load has been shown to moderate interference caused by supraliminal (consciously perceived) emotional stimuli [3] and to down-regulate activity in emotion processing centres (e.g., [10]). This suggests that stimulus salience and task difficulty influence the level of attentional resources captured during goal attainment. It is unclear, however, whether this effect of load on emotional processing is reliant on metacognition, i.e., the ability to reflect on or regulate cognitive processes, or whether it occurs in the absence of conscious awareness. This study addresses this question by investigating the effect of masked emotional (appetitive and aversive) and neutral stimuli on a working memory task (n-back) of varying difficulty (1-back and 2-back).

Due to the limited capacity of attentional resources, individuals must attend to some stimuli at the expense of others [4]. Models of selective attention and emotional interference [5]–[9] propose that levels of attention are determined by the balance between ‘bottom-up’ sensory stimulus-driven influences such as salience (e.g., motivational value) and ‘top-down’ goal-directed influences (e.g., task demands). Thus, behaviours may be can be seen as lying on a continuum between reflexive and voluntary, and depends on the relative influence of these two factors [5]. For example, a biased competition model [8] proposes that attentional resources are *biased* towards salient stimuli, but are also driven by top-down regulation or feedback. In addition, individual differences in state, trait or personal relevance of the goal or distracter stimuli are thought to interact with stimulus salience and task demands to contribute to the biasing of attentional allocation (for rev., see [9], [11]). Support for such models is provided by demonstrations of interference, e.g., in terms of slowed response time or greater errors on trials accompanied by additional emotional (vs. neutral) information during affective variations of executive tasks (e.g., [12]–[15]). In an affective variation of the Stroop task, individuals are slower to name the colour of words with an emotional meaning (e.g., [16]). Similarly, response times on the n-back task are slower when targets are accompanied by emotional versus neutral words [17], [18]. Greater interference from positively and negatively valenced emotional stimuli compared to neutral stimuli has been seen in non-clinical populations [15], [16], while in anxious or depressed groups, negative stimuli elicit the greatest interference [19]. These data support the biased or preferential allocation of attention towards salient emotional compared to neutral stimuli.

In contrast to the frequently reported interference effect, a facilitation effect has also been reported (e.g., [20], [21]): this may be partly determined by the relevance or congruency between emotional stimuli and the cognitive task. Thus, if emotional information is task-relevant or aligned with the task's emotional context, performance is likely to improve as resources needed to process the emotional information will also be devoted to the task (e.g., [20]), but if it is task-irrelevant or incongruent with the task stimuli, resources allocated to its processing will be unavailable to the task and interference will result [9]. This suggests that when there is competition for limited attentional resources, emotional information will be preferentially processed over non-emotional information [4]. Thus, the influence of emotional stimuli on cognitive performance as a consequence of attentional allocation is at least partly dependent on task relevance.

Additionally, goal-directed influences may impact the level of attentional resources captured by task relevant and irrelevant stimuli. It has been proposed that increasing the difficulty of goal attainment is associated with greater motivational arousal, as reflected in greater subjective and physiological levels of arousal (e.g., [22]). Thus, increasing motivational incentive by enhancing task difficulty may bias attentional resources towards the task-relevant stimuli. Moreover, more difficult tasks require greater concentration, thereby occupying greater attentional resources. If competition for attentional resources between cognitive and emotional processing exists, then interference by irrelevant emotional stimuli will be sensitive to cognitive load, i.e., increasing cognitive load would leave less attentional resources available for processing task-irrelevant emotional stimuli. As a consequence, interference should be reduced as task demands increase. There is support for this, i.e., that emotional interference effects are affected by cognitive load due to competition for attentional resources For example, research has shown that task-irrelevant emotional expressions interfere with performance on gender naming tasks during low but not high working memory load [3], [23]. These findings are supported by neuroimaging data showing decreased activity in emotion processing centres under conditions of increased load [10], [14], [24]–[26], although note that in the study by Erk et al. [25], emotional distracters had no behavioural effect on task performance. Such load-related downregulation has even been observed in the absence of emotional distracters [26]. Thus, biased processing of salient emotional stimuli appears to be dependent on the availability of attentional resources. If such competition does not exist, attentional resources should remain biased towards processing of salient stimuli regardless of their relevance to the task, and thus interference should be seen at all levels of task difficulty. It has been proposed that load-dependent elimination of interference may be specific to perceptual load, but does not generalise to cognitive load [27]. If perceptual and top-down cognitive processes do not compete for the same attentional resources, increasing cognitive load would exhaust resources available for cognitive processing, leaving less for actively regulating selective attention and maintaining goal-directed behaviour [28]. Greater load may therefore enhance interference caused by emotional stimuli [29], [30]. Accordingly, greater interference has been associated with increased load (e.g., [29]). For example, Lavie and de Fockert [30] reported that performance of a visual search task was impaired under conditions of high working memory load compared to no or low working memory load; however, the distracter stimuli had goal-relevant features, even though they did not provide task-relevant information.

Many studies on the effects of emotional stimuli on cognition are ‘implicit’, i.e., stimuli are available for cognitive processing but not fully attended to. These include presenting emotional stimuli as background images (e.g., [19], [31]) or flanking stimuli (e.g., [18]) in an emotional n-back paradigm, or including emotionally expressive faces in a gender naming task [3], [23]. However, as the emotional aspect of these stimuli remains available for conscious processing and evaluation, responses may be influenced by metacognition, i.e., the ability to reflect on and regulate one's cognitive activity [32], [33]. However, subliminally presented emotional stimuli (e.g., using masking paradigms) have been shown to affect behaviour (e.g., [34]) and activity in emotion processing centres, including the amygdala [35]. Interestingly, the effects of subliminal stimuli on behaviour are distinguishable from those caused by supraliminal stimuli (for rev., see [2]). For example, one study reported that priming (modulating behaviour as a function of exposure to an emotional stimulus) with emotional faces only affected subjects' ratings of their impression of four targets when the primes were presented subliminally, suggesting that individuals can control their bias to supraliminal primes [36]. Neuroimaging data are in accord with these behavioural studies, suggesting that processing of subliminal and supraliminal emotional stimuli involve different neural substrates (e.g, [37], [38]). For example, Liddell et al. [39] observed a double dissociation in an ERP study comparing perception of subliminal vs. supraliminal fearful and neutral stimuli, with subliminal fear perception associated with enhancement of early components thought to be related to “orienting”, and supraliminal fear being associated with enhancement of later components involved in “event integration”. They proposed these findings reflect the time course of fear perception: initial automatic, non-conscious processing, followed by later controlled, evaluative conscious processing. From such data, it has been suggested that supraliminal stimuli activate post-attentive metacognitions to regulate or suppress pre-attentive processing biases [40]. Lack of awareness of stimuli may therefore impede metacognitive regulation [41]. It is therefore of interest to examine how top-down goal-directed influences affect the automatic bias towards salient stimuli during a cognitive task in the absence of metacognitions.

To maintain efficient neural processing, goal-directed cognitions and salient irrelevant stimuli often compete for attention outside of conscious awareness. Given the data supporting the influence of subliminal emotional stimuli on behaviour, it is likely that unaware stimuli can capture attentional resources, but the extent to which this occurs is unclear. It is also likely that competitive interference exists outside of conscious awareness. If this is the case, subliminal stimuli will interact with cognitive load in the same way as supraliminal stimuli, i.e., interference from irrelevant emotional stimuli will occur under low, but not high load. On the other hand, if top-down goal-directed cognitive regulation requires the assistance of metacognitive processes, regulation of biases towards salient stimuli will not occur, and interference will be expected at both high and low load.

In the present study, we used masking to investigate the effect of subliminal irrelevant emotional stimuli on performance of a cognitive task (n-back) at low load (1-back) and high load (2-back). We also explored the generalisability of interference effects by using multiple categories of salient emotional stimuli with high motivational value (appetitive and aversive images). As cognition-emotion interactions occur outside of awareness, goal-directed influences are predicted to regulate emotional processing even in the absence of conscious awareness. Thus, it is hypothesised that (a) greater interference will result from presentation of masked emotional compared to neutral stimuli; (b) greater interference will occur under conditions of low compared to high cognitive load; (c) there will be no difference in interference elicited by the two types of emotional stimuli (appetitive and aversive).

## Methods

### Ethics statement

The study was approved by the Institute of Psychiatry, KCL ethics committee (297/02). The study adhered to the guidelines as set out in the Declaration of Helsinki. All participants gave written consent after the procedures were explained and were debriefed after the experiment. Participants were reimbursed £10 for their time.

### Participants

31 healthy participants (18 female) were recruited by email advertisement at King's College London. Their mean age was 25.4 years (SD 8.7; range 18–55) and they had a mean of 15.4 (SD 2.3; range 10–20) years of formal education. Mean IQ, estimated by the National Adult Reading Test (NART; [42]), was 114.0 (SD 8.9; range 91–127). All were native English speakers. Exclusion criteria: axis I mental disorder, neurological disease, history of head trauma with loss of consciousness and current use of psychotropic medication. Participants were forewarned that they may be presented with emotionally strong images but not told these would be embedded in cognitive tasks.

### Procedure

**Figure 1:**
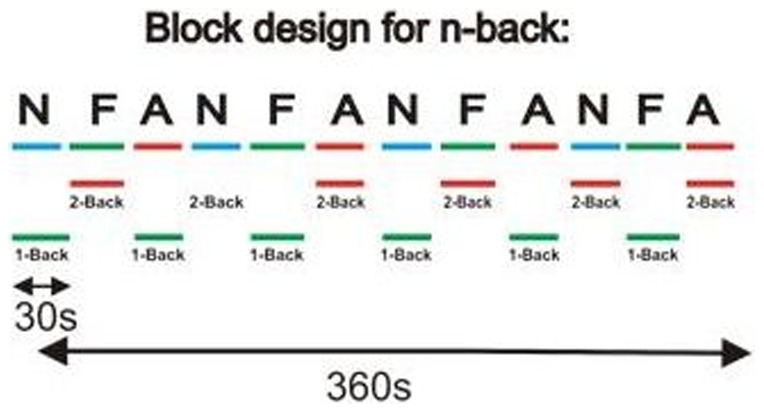
Paradigm design. This figure illustrates the block design for the N-back task, in which the type of masked distracter stimuli (NEUTRAL (N); FOOD (F); AVERSIVE (A)) and the level of difficulty (1-BACK, 2-BACK) are alternated in a fixed order with each 30 s block. A total of 12 blocks are presented in the study, with 20 trials (involving presentation of 20 distracter stimuli and 20 letter targets) in each block.

After screening using the Structured Clinical Interview for Diagnosis (SCID), Researcher Version [43], participants performed the computerised n-back task involving two levels of difficulty (1-back, 2-back). A block design was employed, with each block comprised of 20 trials. On each trial, a distracter stimulus was presented briefly, followed by presentation of an n-back target on a mosaic background. Thus, 20 distracter stimuli (of the same distracter type) and 20 n-back targets were presented in each block. All trials within a block were of the same level of difficulty (1-back or 2-back) and distracters were of the same distracter type (aversive, appetitive, or neutral). The task design is illustrated in figure 1. Difficulty level and distracter type alternated with each block in a fixed order, which was counterbalanced between participants to control for order effects and differential transfer. Each distracter stimulus was used 4 times throughout the task, with distracters presented in a pseudorandomised order.

**Figure 2:**
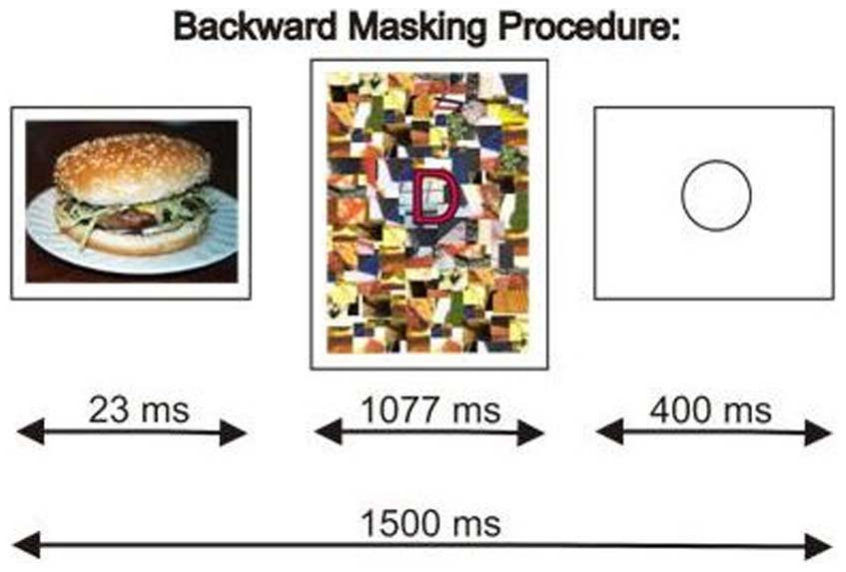
Schematic diagram of the backward masking procedure. This figure illustrates the order and timing of presentation of distracters and targets, using the example of a food stimulus (presented for 23 ms so as to not be consciously perceived), immediately followed by a target letter (in this example, ‘D’) on a mosaic background for 1077 ms, followed by a blank fixation circle on a white background for 400 ms.

The influence of conscious processing of the distracters was minimised by using a backward masking procedure [44]. Distracters (12×8 cm) were presented on a screen for 23 ms, immediately preceding each target. Pilot data using the same apparatus and stimuli indicated that a stimulus onset asynchrony (SOA) of 23 ms was ideal to reliably present stimuli on the screen (all stimuli are visible in the absence of mask) and prevent awareness of stimuli in most subjects. In both tasks, participants were presented with a sequence of letters as targets (2.4 cm high, in red) on a background of a high contrast mosaic (eight mosaics used in a random order), which served as a mask for backward masking of the stimuli. Each letter was presented for 1077 ms and followed by a blank screen for 400 ms. The resulting rate of presentation was one screen every 1.5 sec. The backward masking procedure used in this study is illustrated in figure 2.

After both tasks, a forced choice test assessed the effectiveness of the masking procedure [44]. Tasks were presented on a 15″ computer screen at eye level at a distance of 50 cm. Responses were made using a computer mouse.

### Distracter stimuli

Colour images of appetitive foods (e.g. cake) and aversive images (e.g. a bloody body) were used as positively and negatively valenced stimuli, respectively. Appetitive, aversive and neutral visual stimuli were selected in two steps. In the first step, a collection of images for each category were preselected to provide a diversity of content and minimise overlap between categories, with attention paid to clarity and recognisability. We also sought to minimise the cultural dependence of the content and meaning of stimuli. Stimuli were selected from 56 colour photographs that were preselected from the International Affective Picture System (IAPS; [45]) and 126 images of items presented on a white plate on a blue background from a database created by the authors (stimuli available upon request). In the second step, five volunteers rated all preselected photographs for pleasantness, aversion, salience, visual complexity and recognisability using a computerised Visual Analogue Scale (1–100). Based on these ratings, 20 from each category were selected according to the following equally weighted criteria: 1) recognisability (unambiguous content, easy to recognise); 2) maximum aversion (aversive stimuli); maximum pleasantness (appetitive stimuli); 3) categories matched for complexity and colour.

### Appetitive stimuli

Colour photographs of palatable sweet (e.g. chocolate) and savoury (e.g. pasta) foods were selected from the IAPS; additional photographs were created by the authors and matched to neutral pictures for colour and visual complexity. Average ratings (max = 100) were: salience 61 (SD 13), pleasantness 71 (SD 10), aversion 19 (SD 11), complexity 34 (SD 17), recognisability 82 (SD 13).

### Aversive stimuli

The IAPS photographs included scenes that elicit withdrawal-motivated emotional states (e.g., violence). Average ratings were: salience 75 (SD 17), pleasantness 30 (SD 9), aversion 68 (SD 8), complexity 48 (SD 23), recognisability 73 (SD 15).

### Neutral stimuli

Colour photographs of neutral inanimate objects (e.g., household objects) were created by the authors. Average ratings were: salience 43 (SD 23), pleasantness 56 (SD 9), aversion 24 (SD 9), complexity 35 (SD 17), recognisability 85 (SD 11).

### Mosaic background images

A high contrast coloured mosaic was used as the backward mask. The mosaic was created by the authors using unrecognisable sections of the distracter stimuli as tiles. This procedure was followed to ensure that the mask and subliminal stimuli shared features (e.g., colour) in order to minimise the threshold at which stimuli became subliminal [46]. Target and non-target letters were presented in the centre of the mosaic (figure 2).

### The N-back task

This task assesses working memory, attention and visuospatial awareness [47]. Demand on working memory can be modified using a different number (n) (1 or 2). To examine the relationship between emotional interference and task load, alternating blocks of 1-back (low working memory load) and 2-back (high working memory load) were used. Participants were presented with a sequence of upper and lower case letters presented in a bold, red colour and asked to respond if the same letter (independent of case) was shown as the one preceding it by ‘n’ positions. Responses were recorded between 100–1500 ms after onset of letter (target) presentation. Blocks of 1-back and 2-back task (20 trials per block) were separated by 4-sec intervals when the inscription ‘1-BACK’ or ‘2-BACK’ was presented on the screen. There were six blocks of the 1-back and six blocks of the 2-back task and in both, 20% of letters were targets. Cross-targets were eliminated, so that there were no 1-back targets in the 2-back task and vice versa. For each task, a short training sequence (20 trials) was used to ensure participants understood instructions. Task difficulty (load) and target letter sequences were counterbalanced between participants (12 permutations of stimulus category and task difficulty were used).

### Forced choice task

After completion of all tasks, participants were asked if they had seen any meaningful images during the tasks. If “yes”, they were asked to provide a written description of what they had seen. In a second step, participants were told about the masked images and shown all stimuli paired with novel stimuli matched for content category. They were asked to choose a picture that they might have seen and guess when uncertain. Both pictures (12×8 cm) were presented adjacent to each other on the screen (position was counterbalanced between stimuli and novel pictures) until the participant responded. Although this task is thought to indicate awareness of stimuli (e.g., [44]), a definite indication of awareness cannot be ascertained due to potential issues related to memory consolidation.

### Statistical Analysis

The dependent variables were the proportion of incorrect responses (error rate) and average time to correct response (response time) at each level of task load. Two separate 3×2 repeated measures analysis of variance (ANOVA) were performed to explore the effects of distracter type (aversive, appetitive, neutral) and task load (1-back, 2-back) on accuracy and on response time in the n-back task.

## Results

### Forced choice performance

#### Awareness of distracter stimuli

Eleven participants reported seeing images during the tasks. In five, descriptions did not match any of the distracter stimuli, and the ‘seen’ images were patterns in the mosaic mask (e.g., crowds, pebbles). These participants typically reported seeing the same image all the time and these were not identified in the forced choice. Three correctly described one image and three described several images: they (4 females, 2 males) were considered ‘aware’ and excluded from analyses. The remaining 25 participants (14 females) performed at chance level in the objective forced choice measure, identifying on average 29.96/60 (SD 4.5) of the stimuli and 30.04/60 (SD 4.5) of novel comparison pictures as seen during the tasks, and were thus included in further analyses.

### N-back task

#### Effects of masked distracters on accuracy

Results from the n-back task were analysed using a 3×2 repeated measures ANOVA, comparing the within-subjects factors of task load (1-back, 2-back) and distracter type (neutral, appetitive, aversive) on mean error rate. As the Mauchly's test of sphericity was non-significant for the main effect of condition (χ^2^(2) = 1.911, p = 0.385), and the load*distracter type interaction (χ^2^(2) = 1.795, p = 0.408), compound sphericity can be assumed in this analysis. This analysis identified a main effect of load, *F*(1,30) = 32.068, *p*<0.001, which appeared to be driven by a higher mean error rate (ME) in the 2-back task for all three distracter types (aversive: ME = 2.42, SD = 2.391; appetitive: ME = 2.39, SD = 2.789; neutral: ME = 2.71, SD = 2.069), compared to the 1-back task (aversive: ME = 1.55, SD = 1.895; appetitive: ME = 1.26, SD = 1.807; neutral: ME = 0.55, SD = 1.060). The mean error rates and associated standard deviations and effect sizes are displayed in table 1. The main effect of distracter type was not significant in this analysis, *F*(3,60) = 1.029, *p* = 0.364. A significant interaction between load and distracter type was observed, *F*(2,60) = 3.528, *p* = 0.036.

**Table 1:** Accuracy and response times in the go/no-go and n-back tasks.

Task	Type of masked distracter	Mean Error ± SD	Effect size^a^	Response time (ms) ± SD	Effect size^a^
**1-BACK**	Aversive	1.55±1.895	0.66**	496.06±90.888	0.17
	Food	1.26±1.807	0.49*	494.83±72.769	0.18
	Neutral	0.55±1.060		483.10±57.187	
**2-BACK**	Aversive	2.42±2.391	0.13	560.37±97.999	0.2
	Food	2.39±2.789	0.13	543.12±93.903	0.03
	Neutral	2.71±2.069		539.46±117.135	

a Effect size relative to neutral, given as Cohen's *d*.

*Trend towards a difference from neutral condition after Bonferroni correction (*p*<0.1).

**Significant differences from the neutral condition after Bonferroni correction (*p*<0.01).

Post-hoc paired-samples t-tests using Bonferroni correction indicated that this interaction was driven by significantly more errors in response to aversive compared to neutral masked stimuli, *t*(30) = 3.288, *p* = 0.015, and a trend for greater errors in response to appetitive stimuli compared to neutral stimuli, t(30) = 2.580, *p* = 0.09, in the 1-back task. No significant differences in accuracy were observed to result from appetitive compared to aversive stimuli in the one-back task, *t*(30) = 0.769, *p*>0.05. Distracters did not appear to affect accuracy in the 2-back task: post-hoc paired samples t-tests did not reveal any significant differences in error between distracter types: aversive vs. neutral, *t*(30) = -0.884, appetitive vs. neutral, *t*(30) = −0.701, aversive vs. appetitive, *t*(30) = 0.088, all *p*>0.05.

#### Effects of masked distracters on response time

Mean response times of correct responses were analysed by a 3×2 repeated measures ANOVA with the within subject factors of task load (1-back, 2-back) and distracter type (neutral, appetitive, aversive). Sphericity is assumed based on non-significant results on Mauchly's test for distracter type (χ^2^(2) = 0.075, p = 0.963), and the load*distracter type interaction (χ^2^(2) = 0.011, p = 0.994). This analysis revealed a significant main effect of load, *F*(1,30) = 23.437, *p*<0.001, but not distracter type, *F*(2,60) = 1.514, *p* = 0.228. The main effect of load was driven by longer mean response times in the 2-back task to all distracter types (aversive: mean RT = 560.37 ms, SD = 98 ms; appetitive: mean RT = 542.12 ms, SD = 93.90 ms; neutral: mean RT = 539.46 ms, SD = 117.13 ms) compared to the 1-back task (aversive: mean RT = 496.06 ms, SD = 93,89 ms; appetitive: mean RT = 494.83 ms, SD = 72.77 ms; neutral: mean RT = 483.10 ms, SD = 57.19 ms). The mean response times and associated standard deviations and effect sizes are displayed in table 1. No significant interaction was observed between task load and distracter type, *F*(2, 60) = 0.386, *p* = 0.681.

### Correlations between interference coefficients

This study tested the hypothesis that the degree of interference elicited by aversive and appetitive stimuli would be similar as both stimulus categories have high motivational value. This was explored by computing a coefficient of interference from aversive and appetitive stimuli for each level of task load (e.g., [errors in aversive – errors in neutral]/[errors in aversive + errors in neutral]). Coefficients were calculated for the interference from the appetitive stimuli in the same way. The aversive and appetitive interference coefficients were strongly correlated at each level of load after Bonferroni correction for multiple comparisons (1-back: *r* = 0.705, *p* = 0.001; 2-back: *r* = 0.582, *p* = 0.001), but no correlations were found between coefficients of different tasks (e.g., between a 1-back coefficient and a 2-back coefficient), *p*>0.05.

## Discussion

This study explored the impact of task load on interference caused by subliminally presented emotional stimuli. Specifically, it examined whether the load-dependent interference effect observed for supraliminal stimuli occurred when stimuli were not consciously perceived. Our data are consistent with this proposal. Participants made significantly more errors when exposed to masked negative (compared to neutral) images and a trend for more errors after exposure to masked positive (compared to neutral) images in the low load (1-back) condition, but not the high load (2-back) condition. While response times were longer for the high load compared to the low load condition, there was no effect of masked distracter type on response time in either load condition. Thus, masked emotional stimuli impaired accuracy of performance, and this was attenuated by increasing load. This supports our hypotheses that emotional stimuli interfere with performance in the absence of awareness, and that this subliminal interference effect is influenced by task load. The interference observed is unlikely to be due to non-specific distraction as the emotional stimuli were compared with visually similar neutral stimuli. Moreover, strong correlations were found between appetitive and aversive interference coefficients within, but not between, each load condition, supporting our hypothesis that the extent of interference would be similar across emotional stimulus (distracter) categories of high motivational value (appetitive and aversive). Our findings hold several implications. Firstly, the demonstration of interference supports the notion that top-down goal-directed influences and bottom-up sensory/perceptual processes compete for attentional resources. Secondly, interference does not appear to be simply due to conscious evaluation of one's cognitive activity. Thirdly, it indicates that top-down cognitive influences regulate attention in the absence of awareness, i.e., a regulatory mechanism exists that is not dependent on metacognition.

Our findings support the existence of competition between top-down cognitive and bottom-up sensory influences, proposed by numerous models of selective attention [5]–[9]. Although emotional stimuli did not affect response time in our study, we observed load-dependent interference on performance accuracy. The reason for the lack of an observed effect of emotion on response time is not clear, but in fact such differences between the two measures have been observed in other studies. While some studies have observed an effect on response time [15], [19], [40], our findings are consistent with Mitchell et al., [14] who reported that more errors were made in the presence of face stimuli expressing a negative emotion compared to a neutral expression, but that emotional expression had no effect on response time. The observation that load-dependent interference effects occur in response to masked emotional stimuli indicates that selective attention is biased towards salient motivational stimuli, which is regulated by top-down influences even in the absence of explicit awareness. These results complement reports demonstrating that cognitive load reduces interference from supraliminal emotional distracters (e.g., [3], [23]). For example, Van Dillen & Derks [23] reported that high cognitive load eliminated the impaired response time caused by goal-irrelevant emotional distraction. Our data thus extend previous findings by demonstrating that competition between top-down goal-directed influences and salient motivational stimuli occur without awareness and impact behaviour. Moreover, it shows that the interference effect of masked stimuli is load-dependent, suggesting that interactions between cognitive load and emotion do not require metacognitions or awareness of the emotional stimuli. This also argues against the notion that the load-dependent interference effect is specific to perceptual but not cognitive load [27].

Our findings are also in accord with neuroimaging studies exploring emotional processing during cognitive task performance (for rev., see [48]). For example, emotional arousal reduces activity in cortical regions involved in higher level cognition, e.g., prefrontal cortex, and increases activity in emotion processing centres, e.g., amygdala, under low but not high cognitive load [49], [50], suggesting there is a threshold at which behaviour is not influenced by low level stimuli. Moreover, some research groups have even reported that task load down-regulated neural activity at emotional processing sites in response to emotional stimuli [10], [14], [24]–[26] and reduced negative emotions subjectively reported in response to negative stimuli [24]. Future studies may investigate the neural correlates underlying the load*distracter type interaction on accuracy (seen in the present study) to determine whether they are similar to the interference effect seen in studies involving supraliminal stimuli.

The ability to respond rapidly to motivational cues, e.g., those related to threat, food or sex, is essential for survival of a species. Most studies investigating interactions between cognition and emotion have used aversive stimuli, e.g., negative facial expressions or threat-related words/images. Some studies have included a positive stimulus category, often comprised of happy faces, non-threatening animals and evocative words/images, but these are often largely heterogeneous and do not have the same motivational value as the stimuli in the aversive categories. As motivational drives may influence cognition, we sought to establish whether interference effects were consistent across different stimulus categories of high motivational value [9]. Food is a primary reward, is primarily appetitive and generates an approach-motivated emotional state [51]. Food stimuli have been used in a positive valence category (e.g., [19]) and are reportedly more efficient than other positive emotional stimuli at inhibiting acoustic startle responses [52]. Attentional bias towards food has been demonstrated in non-patient populations who are hungry or fasted [53], [54], or are sensitive to external food cues [55], however has mainly been studied in the context of disordered eating (for rev., see [56]). In the present study, interference resulted from exposure to both appetitive and aversive stimuli, suggesting the load-dependent interference effect is not valence specific. Moreover, strong correlations between the appetitive and aversive interference coefficients suggest that the degree of interference is similar across distracter categories. These correlations were only strong within each level of task load, but not between load conditions, i.e., the appetitive and aversive coefficients for the 1-back task correlated with each other, but not with the coefficients calculated for the 2-back task. Our results are consistent with studies showing that emotional stimuli, regardless of valence, influence cognitive processing [12], [16], [57], [58], although there is also evidence for differential effects of valence on performance [59]. However, post-hoc t-tests for the 1-back task indicated that after correction for multiple comparisons, exposure to aversive images elicited significantly more errors compared to neutral distracters, whereas a trend for greater errors after viewing masked positive compared to neutral images was observed. This suggests that while both categories appear to interfere with cognitive performance at low load, and this interference effect is strongly correlated between motivational stimulus categories, the effect is stronger for aversive stimuli.

In addition to task load and stimulus salience, other factors may affect interactions between top-down cognitive and bottom-up sensory processing, e.g., the emotional intensity of salient, task-irrelevant stimuli [60], or individual differences in task motivation and stimulus relevance [11]. One study manipulated the emotional intensity of task-irrelevant stimuli and reported that the attenuation of interference under high cognitive load only occurred when the emotional intensity of the distracting stimuli was high [60]. This is consistent with our data that irrelevant emotional, but not neutral stimuli cause interference at low, but not high cognitive load. However, as emotional intensity was not directly manipulated in this study, it would be interesting to investigate the influence of such additional factors on the load-dependent interference effects using subliminal and supraliminal task-irrelevant stimuli.

This study has some limitations. It is difficult to be certain that masked images were not seen. However, we used an established procedure for masking pictorial stimuli and at the end of the experiment, we ran subjective and objective assessments to ensure images were not consciously perceived. Nonetheless, some masked images may have been seen despite chance performance on the forced choice task. To address this, we combined the forced choice test with some subjective written questions about whether participants had seen any images during the study. Use of positive stimuli might be problematic, given that individual differences in attractiveness ratings are reported to be greater than in aversion ratings [61]. However, we used images of food, which reliably elicit appetitive arousal and can be considered primary emotional stimuli [51]. Although there are individual differences in food preferences, we chose images with consistently positive ratings.

In conclusion, masked emotional (appetitive and aversive), but not neutral, stimuli interfered with performance on a cognitive task at low (1-back), but not high (2-back) load. This is in agreement with reports that supraliminal emotional stimuli exert load-dependent interference effects on performance. Our study extends these findings by demonstrating that this load-dependent interference effect applies to masked emotional stimuli and generalises across stimulus categories with high motivational value. Our data support models of selective attention that propose cognition and emotion compete for attentional resources. Interference from masked emotional stimuli at low load supports the notion that attention is biased towards salient stimuli, while the dissipation of interference under high load indicates the involvement of top-down regulation of attention. As stimuli were not consciously perceived, our data also suggest that such top-down goal-directed regulation of attention may occur in the absence of explicit awareness and hence does not require metacognitive monitoring or evaluation of bias over behaviour. Thus, some degree of self-regulation occurs at a sub-conscious level. Future neuroimaging studies will be required to further delineate the neural mechanisms underlying this effect, and to evaluate the similarity with the neural activity changes reported in response to supraliminal distracters.

## References

[1] Bechara A, Damasio H, Tranel D, Damasio AR (1997) Deciding advantageously before knowing the advantageous strategy. Science 275: 1293–1295. 9036851 10.1126/science.275.5304.1293

[2] Merikle PM, Daneman M (1998) Psychological investigations of unconscious perception. J Consciousness Stud 5: 5–18.

[3] Van Dillen LF, Koole SL (2009) How automatic is “automatic vigilance”? The role of working memory in attentional interference of negative information. Cognition Emotion 23: 1106–1117.

[4] Vuilleumier P (2005) How brains beware: neural mechanisms of emotional attention. Trends Cogn Sci 9: 585–594. 16289871 10.1016/j.tics.2005.10.011

[5] Pashler H, Johnston JC, Ruthruff E (2001) Attention and performance. Annu Rev Psychol 52: 629–651. 11148320 10.1146/annurev.psych.52.1.629

[6] Lavie N (2000) Selective attention and cognitive control: Dissociating attentional functions through different types of load. In: Monsell S, Driver J, editors. Control of Cognitive Processes: Attention and Performance XVIII. Cambridge, Massachusetts: MIT Press.

[7] Yantis S (2000) Goal-directed and stimulus-driven determinants of attentional control. Attention Perform 18: 73–103.

[8] Desimone R, Duncan J (1995) Neural mechanisms of selective visual attention. Annu Rev Neurosci 18: 193–222. 7605061 10.1146/annurev.ne.18.030195.001205

[9] Pessoa L (2009) How do emotion and motivation direct executive control? Trends Cogn Sci 13: 160–166. 19285913 10.1016/j.tics.2009.01.006 PMC2773442

[10] Pessoa L, McKenna M, Gutierrez E, Ungerleider LG (2002) Neural processing of emotional faces requires attention. Proc Natl Acad Sci U S A 99: 11458–11463. 12177449 10.1073/pnas.172403899 PMC123278

[11] Oliveira L, Mocaiber I, David IA, Erthal F, Volchan E, et al (2013) Emotion and attention interaction: a trade-off between stimuli relevance, motivation and individual differences. Front Hum Neurosci 7: 364. 23874284 10.3389/fnhum.2013.00364 PMC3709171

[12] Blair KS, Smith BW, Mitchell DGV, Morton J, Vythilingam M, et al (2007) Modulation of emotion by cognition and cognition by emotion. NeuroImage 35: 430–440. 17239620 10.1016/j.neuroimage.2006.11.048 PMC1862681

[13] Hartikainen K, Ogawa K, Knight R (2000) Transient interference of right hemispheric function due to automatic emotional processing. Neuropsychologia 38: 1576–1580. 11074080 10.1016/s0028-3932(00)00072-5

[14] Mitchell D, Nakic M, Fridberg D, Kamel N, Pine D, et al (2007) The impact of processing load on emotion. Neuroimage 34: 1299–1309. 17161627 10.1016/j.neuroimage.2006.10.012 PMC1909754

[15] Mitchell D, Luo Q, Mondillo K, Vythilingam M, Finger E, et al (2008) The interference of operant task performance by emotional distracters: an antagonistic relationship between the amygdala and frontoparietal cortices. NeuroImage 40: 859–868. 18234519 10.1016/j.neuroimage.2007.08.002 PMC2693278

[16] Dresler T, Mériau K, Heekeren H, Meer E (2009) Emotional Stroop task: effect of word arousal and subject anxiety on emotional interference. Psychol Res 73: 364–371. 18636272 10.1007/s00426-008-0154-6

[17] Bertocci MA, Bebko GM, Mullin BC, Langenecker SA, Ladouceur CD, et al (2012) Abnormal anterior cingulate cortical activity during emotional n-back task performance distinguishes bipolar from unipolar depressed females. Psychol Med 42: 1417–1428. 22099606 10.1017/S003329171100242X PMC3601380

[18] Ladouceur CD, Silk JS, Dahl RE, Ostapenko L, Kronhaus DM, et al (2009) Fearful faces influence attentional control processes in anxious youth and adults. Emotion 9: 855–864. 20001128 10.1037/a0017747

[19] Ladouceur CD, Dahl RE, Williamson DE, Birmaher B, Ryan ND, et al (2005) Altered emotional processing in pediatric anxiety, depression, and comorbid anxiety-depression. J Abnorm Child Psych 33: 165–177. 10.1007/s10802-005-1825-z 15839495

[20] Anderson AK (2005) Affective influences on the attentional dynamics supporting awareness. J Exp Psychol Gen 134: 258. 15869349 10.1037/0096-3445.134.2.258

[21] Levens SM, Phelps EA (2008) Emotion processing effects on interference resolution in working memory. Emotion 8: 267. 18410200 10.1037/1528-3542.8.2.267

[22] Wright RA, Brehm JW (1984) The impact of task difficulty upon perceptions of arousal and goal attractiveness in an avoidance paradigm. Motiv Emotion 8: 171–181.

[23] Van Dillen LF, Derks B (2012) Working memory load reduces facilitated processing of threatening faces: An ERP study. Emotion 12: 1340. 22642340 10.1037/a0028624

[24] Van Dillen LF, Heslenfeld DJ, Koole SL (2009) Tuning down the emotional brain: an fMRI study of the effects of cognitive load on the processing of affective images. NeuroImage 45: 1212–1219. 19349235 10.1016/j.neuroimage.2009.01.016

[25] Erk S, Kleczar A, Walter H (2007) Valence-specific regulation effects in a working memory task with emotional context. NeuroImage 37: 623–632. 17570686 10.1016/j.neuroimage.2007.05.006

[26] Pessoa L, Padmala S, Morland T (2005) Fate of unattended fearful faces in the amygdala is determined by both attentional resources and cognitive modulation. NeuroImage 28: 249–255. 15993624 10.1016/j.neuroimage.2005.05.048 PMC2427145

[27] Lavie N (2005) Distracted and confused?: selective attention under load. Trends Cogn Sci 9: 75–82. 15668100 10.1016/j.tics.2004.12.004

[28] Lavie N, Hirst A, de Fockert JW, Viding E (2004) Load theory of selective attention and cognitive control. J Exp Psychol Gen 133: 339. 15355143 10.1037/0096-3445.133.3.339

[29] de Fockert JW, Rees G, Frith CD, Lavie N (2001) The role of working memory in visual selective attention. Science 291: 1803–1806. 11230699 10.1126/science.1056496

[30] Lavie N, de Fockert J (2006) Frontal control of attentional capture in visual search. Vis Cogn 14: 863–876.

[31] Gläscher J, Adolphs R (2003) Processing of the arousal of subliminal and supraliminal emotional stimuli by the human amygdala. J Neurosci 23: 10274–10282. 14614086 10.1523/JNEUROSCI.23-32-10274.2003 PMC6741000

[32] Fernandez-Duque D, Baird JA, Posner MI (2000) Executive Attention and Metacognitive Regulation. Consciousness Cogn 9: 288–307. 10.1006/ccog.2000.0447 10924249

[33] Charles L, Opstal Fv, Marti S, Dehaene S (2013) Distinct brain mechanisms for conscious versus subliminal error detection. NeuroImage. 10.1016/j.neuroimage.2013.01.054 PMC5635965 23380166

[34] Winkielman P, Berridge KC, Wilbarger JL (2005) Unconscious affective reactions to masked happy versus angry faces influence consumption behavior and judgments of value. Pers Soc Psychol B 31: 121–135. 10.1177/0146167204271309 15574667

[35] Whalen PJ, Rauch SL, Etcoff NL, McInerney SC, Lee MB, et al (1998) Masked presentations of emotional facial expressions modulate amygdala activity without explicit knowledge. J Neurosci 18: 411–418. 9412517 10.1523/JNEUROSCI.18-01-00411.1998 PMC6793390

[36] Mori T, Sakamoto A (1997) The effects of subliminal and supraliminal presentation of trait-related words on person perception. Japan J Psychol 68: 371. 10.4992/jjpsy.68.371 9551539

[37] Pessoa L, Japee S, Sturman D, Ungerleider LG (2006) Target visibility and visual awareness modulate amygdala responses to fearful faces. Cereb Cortex 16: 366–375. 15930371 10.1093/cercor/bhi115

[38] Williams LM, Liddell BJ, Kemp AH, Bryant RA, Meares RA, et al (2006) Amygdala–prefrontal dissociation of subliminal and supraliminal fear. Hum Brain Mapp 27: 652–661. 16281289 10.1002/hbm.20208 PMC6871444

[39] Liddell BJ, Williams LM, Rathjen J, Shevrin H, Gordon E (2004) A temporal dissociation of subliminal versus supraliminal fear perception: an event-related potential study. J Cognitive Neurosci 16: 479–486. 10.1162/089892904322926809 15072682

[40] Mogg K, Kentish J, Bradley BP (1993) Effects of anxiety and awareness on colour-identification latencies for emotional words. Behav Res Ther 31: 559–567. 8347114 10.1016/0005-7967(93)90107-6

[41] Skjærvø I (2010) Attentional bias towards supraliminal and subliminal smoking cues in smokers: Effects of cognitive load on attentional bias: University of Oslo.

[42] Nelson HE, Willison J (1991) National Adult Reading Test Manual. Windsor: NFER-Nelson.

[43] First MB, Spitzer RL, Gibbon M, Williams JBW (2002) Structured Clinical Interview for DSM-IV-TR Axis I Disorders, Research Version, Non-patient Edition (SCID-I/NP). New York: Biometrics Research, New York State Psychiatric Institute.

[44] Esteves F, Öhman A (1993) Masking the face: Recognition of emotional facial expressions as a function of the parameters of backward masking. Scand J Psychol 34: 1–18. 8322040 10.1111/j.1467-9450.1993.tb01096.x

[45] Lang PJ, Bradley MM, Cuthbert BN (1996) International Affective Picture System: IAPS. New York,: NIMH Centre for the Study of Emotion and Affect.

[46] Turvey MT (1973) On peripheral and central processes in vision: inferences from an information-processing analysis of masking with patterned stimuli. Psychol Rev 80: 1. 4689202 10.1037/h0033872

[47] Andrews SC, Hoy KE, Enticott PG, Daskalakis ZJ, Fitzgerald PB (2011) Improving working memory: the effect of combining cognitive activity and anodal transcranial direct current stimulation to the left dorsolateral prefrontal cortex. Brain Stimul 4: 84–89. 21511208 10.1016/j.brs.2010.06.004

[48] Drevets WC, Raichle ME (1998) Reciprocal suppression of regional cerebral blood flow during emotional versus higher cognitive processes: Implications for interactions between emotion and cognition. Cognition Emotion 12: 353–385.

[49] Hart SJ, Green SR, Casp M, Belger A (2010) Emotional priming effects during Stroop task performance. NeuroImage 49: 2662–2670. 19883772 10.1016/j.neuroimage.2009.10.076 PMC2818423

[50] Clarke R, Johnstone T (2013) Prefrontal inhibition of threat processing reduces working memory interference. Front Hum Neurosci 7.. 10.3389/fnhum.2013.00228 PMC3667546 23750133

[51] Rolls ET (1999) Brain and emotions. Oxford: Oxford University Press.

[52] Drobes DJ, Miller EJ, Hillman CH, Bradley MM, Cuthbert BN, et al (2001) Food deprivation and emotional reactions to food cues: Implications for eating disorders. Biol Psychol 57: 153–177. 11454438 10.1016/s0301-0511(01)00093-x

[53] Castellanos EH, Charboneau E, Dietrich MS, Park S, Bradley BP, et al (2009) Obese adults have visual attention bias for food cue images: evidence for altered reward system function. Int J Obesity 33: 1063–1073. 10.1038/ijo.2009.138 19621020

[54] Mogg K, Bradley BP, Hyare H, Lee S (1998) Selective attention to food-related stimuli in hunger: are attentional biases specific to emotional and psychopathological states, or are they also found in normal drive states? Behav Res Ther 36: 227–237. 9613028 10.1016/s0005-7967(97)00062-4

[55] Brignell C, Griffiths T, Bradley BP, Mogg K (2009) Attentional and approach biases for pictorial food cues. Influence of external eating. Appetite 52: 299–306. 19027808 10.1016/j.appet.2008.10.007

[56] Lee M, Shafran R (2004) Information processing biases in eating disorders. Clin Psychol Rev 24: 215–238. 15081517 10.1016/j.cpr.2003.10.004

[57] de Jong PJ, van den Hout MA, Rietbroek H, Huiding J (2003) Dissociation between implicit and explicit attitudes towards phobic stimuli. Cognition Emotion 17: 521–545. 10.1080/02699930302305 29715728

[58] Dalgleish T, Taghavi R, Neshat-Doost H, Moradi A, Canterbury R, et al (2003) Patterns of processing bias for emotional information across clinical disorders: A comparison of attention, memory, and prospective cognition in children and adolescents with depression, generalized anxiety, and posttraumatic stress disorder. J Clin Child Adolesc 32: 10–21. 10.1207/S15374424JCCP3201_02 12573928

[59] Eder AB, Rothermund K (2010) Automatic influence of arousal information on evaluative processing: Valence–arousal interactions in an affective Simon task. Cognition Emotion 24: 1053–1061.

[60] Shafer AT, Matveychuk D, Penney T, O'Hare AJ, Stokes J, et al (2012) Processing of emotional distraction is both automatic and modulated by attention: evidence from an event-related fMRI investigation. J Cognitive Neurosci 24: 1233–1252. 10.1162/jocn_a_00206 PMC4491634 22332805

[61] Hermans D, Spruyt A, De Houwer J, Eelen P (2003) Affective priming with subliminally presented pictures. Can J Exp Psychol 57: 97. 12822839 10.1037/h0087416

